# Cell-type-specific differences in KDEL receptor clustering in mammalian cells

**DOI:** 10.1371/journal.pone.0235864

**Published:** 2020-07-09

**Authors:** Achim Bauer, Ludger Santen, Manfred J. Schmitt, M. Reza Shaebani, Björn Becker

**Affiliations:** 1 Molecular and Cell Biology, Department of Biosciences and Center of Human and Molecular Biology (ZHMB), Saarland University, Saarbrücken, Germany; 2 Department of Theoretical Physics and Center for Biophysics, Saarland University, Saarbrücken, Germany; The University of British Columbia Life Sciences Institute, CANADA

## Abstract

In eukaryotic cells, KDEL receptors (KDELRs) facilitate the retrieval of endoplasmic reticulum (ER) luminal proteins from the Golgi compartment back to the ER. Apart from the well-documented retention function, recent findings reveal that the cellular KDELRs have more complex roles, e.g. in cell signalling, protein secretion, cell adhesion and tumorigenesis. Furthermore, several studies suggest that a sub-population of KDELRs is located at the cell surface, where they could form and internalize KDELR/cargo clusters after K/HDEL-ligand binding. However, so far it has been unclear whether there are species- or cell-type-specific differences in KDELR clustering. By comparing ligand-induced KDELR clustering in different mouse and human cell lines via live cell imaging, we show that macrophage cell lines from both species do not develop any clusters. Using RT-qPCR experiments and numerical analysis, we address the role of KDELR expression as well as endocytosis and exocytosis rates on the receptor clustering at the plasma membrane and discuss how the efficiency of directed transport to preferred docking sites on the membrane influences the exponent of the power-law distribution of the cluster size.

## Introduction

Recent discoveries in the KDEL receptor (KDELR) research field have strongly changed the common understanding of the role of these fascinating transmembrane proteins. It is obvious now that the three KDELR homologues have more diverse and fundamental isoform-specific roles in eukaryotic systems than previously assumed [1, 2]; KDELRs do not merely maintain the composition of the endoplasmic reticulum (ER) by returning ER-resident proteins from the Golgi into the ER via a pH-dependent retrieval mechanism [3–6]. Under stress conditions, KDELR2 and KDELR3 expression is upregulated on the transcriptional level via the XBP1/IRE1 pathway, one of the three major unfolded protein response pathways in mammalian cells, to counteract the loss of ER-resident proteins [7, 8]. Previous studies also indicated that KDELRs regulate Golgi homeostasis as well as protein secretion by interacting with a subset of different G-proteins at the Golgi membrane [9–11]. After KDELR/ligand interaction in the Golgi lumen, the active G*α* subunits activate their specific target protein kinases (e.g. Src-kinases or PKA), which subsequently modulate gene transcription followed by regulation of the anterograde or retrograde trafficking [9, 10, 12]. The regulation of protein secretion is mediated by a cellular mechanism, called “traffic-induced degradation response for secretion” (TIDeRS), which activates KDELR1-dependent PKA signalling and results in a complex interplay between the cytoskeleton, autophagy and secretion machinery including lysosome relocation as well as autophagy-dependent lipid-droplet turnover [11]. KDELR malfunctions are associated with changes in extracellular matrix degradation and cellular adhesion [13–15]. Recent studies have revealed that upregulated KDELR2 expression level in glioblastoma tissues promotes the tumorigenesis and shortens the lifetime, making the receptor an interesting rapeutic target in glioblastoma patients [16].

It is known that a subpopulation of KDELRs in mammalian and yeast cells are located at the cell surface [17–19], however, the possible reasons of this plasma membrane (PM) localization are not fully understood. It is suggested that the transport of the ER chaperone isoform PDIA6 to the cell surface depends on its KDEL-motif and is presumably mediated by KDELR1 [20]. Also, PM-localized KDELRs in *S. cerevisiae* serve as specific A/B toxin receptors which are hijacked by the yeast killer toxin K28 to ensure its cell entry [17]. Nevertheless, a more natural role of KDELRs at the yeast cell surface is the reinternalization of mistrafficked ER-resident proteins from the yeast PM to prevent their permanent loss as well as their new synthesis, thus, saving energy and cellular resources [17]. Based on recent studies using mesencephalic astrocyte-derived (MANF) or cerebral dopamine (CDNF) neurotrophic factors [19, 21], it seems that the KDELRs at the cell surface are also involved in cell-cell communication by sensing ER stress level between tissue cells through binding secreted KDELR ligands of stressed neighbouring cells at the PM level. For CDNF and MANF, a neuro- and cardio-protective effect is postulated which is presumably mediated via initial KDEL receptor binding and upstream activation of signalling pathways (e.g. PI3K/AKT for CDNF), similar to KDELR-dependent signalling processes at the Golgi compartment [19, 21]. Cells have surely developed specific mechanisms and/or signalling pathways to respond to ligand binding and to regulate the KDELR expression level at the cell surface, however, the molecular machinery responsible for KDELR transport to the PM has not been well-characterized.

So far, it is known that KDELRs form clusters in HeLa cells in the presence of an artificial model cargo containing a C-terminal ER retention motif (HDEL or KDEL) [18]. It has also been demonstrated that cargo binding induces an increased microtubule-assisted KDELR transport to preferred arrival sites at the PM [18]. However, it is unclear whether cluster formation at the PM is a cell-type- or species-specific process. Here we study KDELR clustering in different mouse and human cell lines by live cell imaging. Our main observation is that mouse and human macrophage cell lines do not show any KDELR cluster formation at the cell surface after ligand treatment. Additionally, the clustering dynamics are qualitatively similar in cell types which develop receptor clusters at the PM, independent of species identity. By means of RT-qPCR experiments, we exclude the possibility that the low mRNA level of KDELRs is responsible for the missing receptor clustering phenotype seen in macrophages. The recycling and cluster formation at cell membranes has more generally attracted attention as a nonequilibrium stochastic process [22, 23]. Here we consider a stochastic KDELR endo/exocytosis model and perform Monte Carlo simulations to better understand how the differences in cluster formation in various cell types may originate from the differences in their endocytosis and/or exocytosis rates.

## Materials and methods

### Cultivation of human and mouse cell lines

HeLa (ATCC number CCL-2), HEK-293T (Invitrogen), SH-SY5Y (Sigma), RAW-Blue (Invitrogen), L929 and MEF cells were cultivated in DMEM medium (Gibco) supplemented with 1% penicillin/streptomycin (PAA) and 10% fetal bovine serum (Biochrom) in a humidified environment at 37°C and 5% CO_2_. IC21 and THP1 (ATCC number TIB-202) were cultivated in RPMI-1640 medium (Gibco) supplemented and cultivated as listed above. To differentiate THP1 cells to macrophages, cells were pre-treated with phorbol 12-myristate 13-acetate (PMA, 30 *μ*g/ml) for 72 h and subsequently used for live cell imaging.

### Production/Purification of KDELR model cargo

Expression of enhanced GFP-tagged RTA variants eGFP-RTA^E177D^ and eGFP-RTA^E177D-HDEL^ in *E. coli* and the subsequent affinity purification procedure was performed as previously described in [18]. Substitution of aspartate for glutamate at position 177 in the model cargo leads to a 50 fold reduction of RTA cytotoxicity [24] and was done by conventional PCR with primers listed in S1 Table.

### Live cell imaging

In imaging experiments, 1.5×10^5^ cells of different cell lines were seeded out in 60*μ*-ibiTreat-dishes (Ibidi) and pre-cultivated for 24 h. Next, the cells were washed two times with PBS (pH 7.4) and cultivated in DMEM (w/o phenol red, 10% FCS) and subsequently analyzed by confocal laser scanning microscopy. To investigate cargo-induced clustering at the PM, cells were treated with 160 *μ*g/ml of eGFP-RTA^E177D-HDEL^ and monitored for 3 h at 37°C and 5% CO_2_. Thereby, the RTA variant lacking a KDELR binding site (eGFP-RTA^E177D^, 160 *μ*g/ml) served as negative control and was monitored for 1.5 h. The time resolution in each experiment is expressed as frames per hour (frames/h).

### Confocal microscopy

Live cell imaging of eGFP-RTA^E177D^ or eGFP-RTA^E177D-HDEL^ was performed by confocal fluorescence microscopy using a Zeiss LSM 510 META (Nikon PlanApo 63x NA 1.4 oil immersion lens, 488 nm excitation, 1.1% argon laser power, HFT 488 and NFT 490 beam splitter, BP 500-530 filter). The same laser power and pinhole size (73 *μ*m) were used to collect all images in each experiment.

### Evaluation of cluster-size distribution

The frames extracted from the experimental videos were converted to gray scale images. Next, an anisotropic Gaussian filter was used to smoothen the intensity field by determining the background noise around each local intensity peak. A threshold ratio between each local peak and its background intensity was used to subtract the noise to obtain the receptor clusters. The chosen parametrization of the smoothening procedure does not qualitatively influence the image analysis results. By converting the pixel gray-scale intensity to a binary array and using the Hoshen-Kopelman algorithm the clusters were identified and by a logarithmic binning of the size range the cluster-size distribution was obtained.

### Gene expression analysis via real time qPCR

For RNA isolation, 1×10^6^ cells were cultivated in the appropriate medium for 24 h in 6-well plates and total cellular RNA was isolated using the Direct-zol RNA MiniPrep Plus Kit (Zymo Research) following the manufacturer’s instructions. Next, 500 ng of RNA was transcribed into cDNA using Maxima Reverse Transcriptase (200 U, Thermo Fisher Scientific), Oligo(dT)18 Primer (100 nM, Thermo Fisher Scientific) and dNTP Mix (each dNTP 0.4 mM, Thermo Fisher Scientific). Finally, a 10 *μ*l qPCR mix was prepared including 2 ng cDNA, 0.2-1 mM of the corresponding primers (see S2 Table) and 2 *μ*l of 5 × Hot-Start-Taq2 qPCR EvaGreen Mix (Axon). RT-qPCR was performed in 40 amplification repeats according to the Hot-Start-Taq2 qPCR EvaGreen manual instructions with primer-optimized annealing temperature using CFX Connect Real-Time System (BioRad). Data analysis was carried out with CFX Manager 3.1 (BioRad).

### Primers, probes and statistical analysis

KDELR primer efficiency was analyzed in a DNA standard curve by a five-log dilution series of either HeLa or L929 cDNA. A no-template control or no-reverse transcriptase control was applied to detect genomic DNA contaminations. Biological replicates (*n* = 3) as well as technical replicates (*n*≥2) were used to determine KDELR gene expression levels. All Cq values were normalized to the mean of the Cq values of the reference gene glyceraldehyde 3-phosphate dehydrogenase (GAPDH) and are represented as a mean for E^-dCq^ (E = primer efficiency) or E^-ddCq^ with error bars representing the upper and lower limits based on the standard deviation of delta Cq values. Statistical analysis was carried out in Graphpad Prism8. All pooled data were given as mean values ± SEM, and statistical significance was assessed by one-way ANOVA based on biological replicates and at sample sizes of *n* = 3.

## Results

### KDELR clustering is cell-type specific and independent of species identity

**Fig 1:**
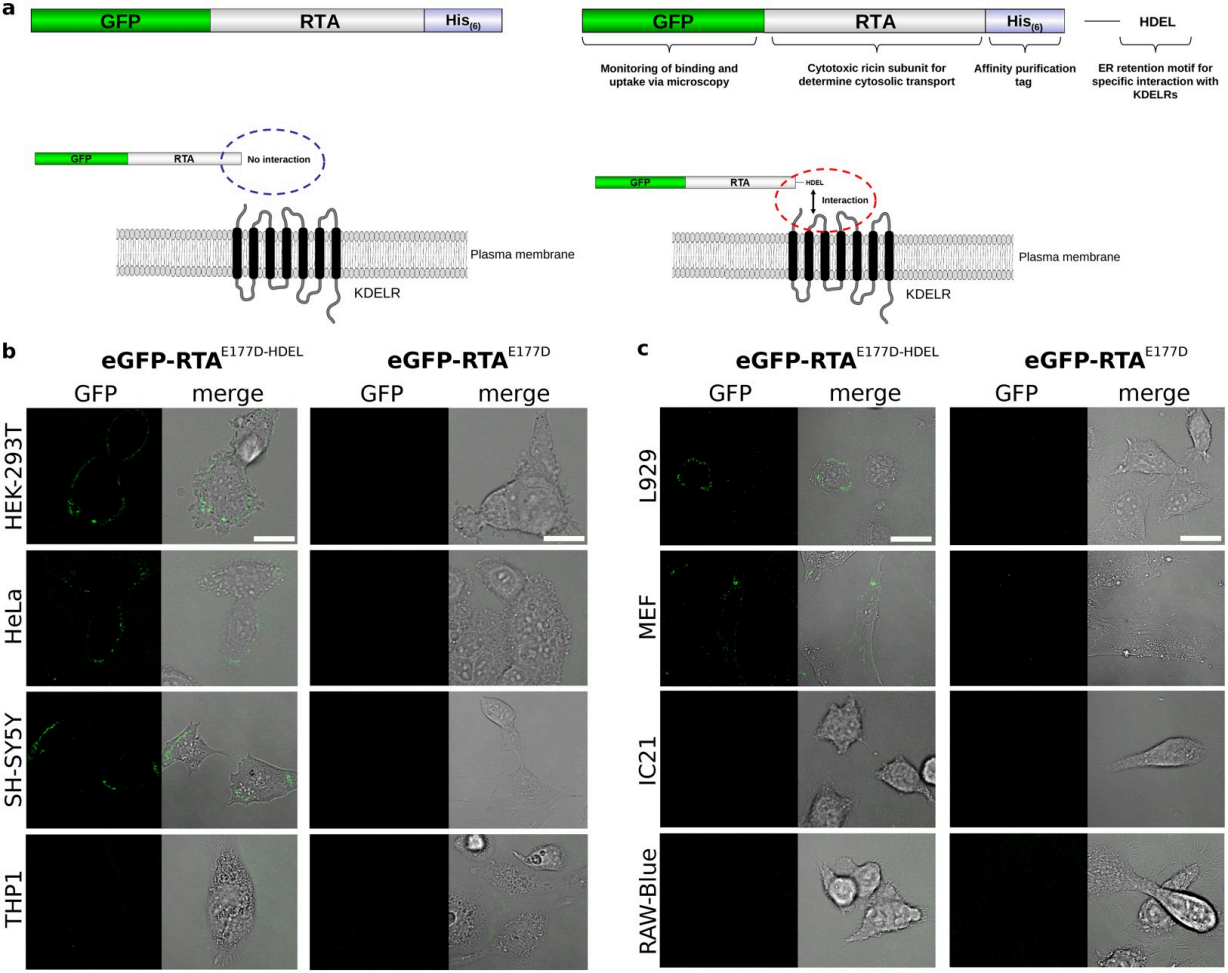
(a) Schematic of the fluorescent model cargo without (top-left, eGFP-RTA^E177D^) or with (top-right, eGFP-RTA^E177D-HDEL^) the ER-retention motif HDEL, which ensures the physical interaction with KDELRs. eGFP-RTA^E177D^ lacking the HDEL motif serves as negative control and is unable to bind KDELRs. An additional mammalian enhanced GFP tag was introduced at the N-terminus to monitor the binding/uptake and interacellular transport of the model cargo in living cells. Furthermore, a less toxic variant of the cytotoxic A subunit of ricin (RTA^E177D^) serves as a second marker to determine the cargo uptake via cell viability. The integrated His-tag ((His)_6_-Tag) is used for affinity purification of the fluorescent model cargos from *E. coli* lysates. (bottom row) Schematics of cargo-KDELR interaction at the mammalian cell surface in the presence/absence of the HDEL motif. (b) Confocal laser scanning microscopy of human cell lines treated with eGFP-RTA^E177D-HDEL^ or eGFP-RTA^E177D^ (negative control). In the latter case, the images represent the steady-state regime of receptor cluster development (*t* ≥ 150 min). Scale bars, 20 *μ*m. (c) Similar to panel (b) but for mouse cell lines.

Previous studies on HeLa cells have demonstrated a ligand-induced KDELR cluster formation at PM-localized KDELR hot spots [18]. In the present work, we perform live cell imaging in various human and mouse cell lines representing different tissues and cell types to determine species- and/or cell-type-specific differences in KDELR cluster formation after external ligand application. For a broad initial comparison, we have chosen to compare cells with epithelial or connetive tissue forming characteristics (human HeLa and HEK-293T; murine L929 and MEF) to non-tissue forming cell types like SH-SY5Y, a human neuronal precurser, and macrophages from both species (human THP1 M0; murine RAW-Blue and IC21) (see S3 Table for the basic characteristics of the cell lines used in this study). In order to minimize the cytotoxicity-induced side effects of the cargo, we replace the wild-type A-subunit of ricin (RTA) of the GFP-tagged model cargo eGFP-RTA and eGFP-RTA^HDEL^ (used in the previous study [18]) with a 50-70-fold less toxic RTA^E177D^ variant [24] (see Fig 1a). Furthermore, we decided to use the ER retention motif HDEL, which is—in addition to KDEL—the most abundant signal in mammalian ER resident proteins [7].

Similar to the experiments with RTA, HeLa cells develop KDEL receptor clusters in the presence of eGFP-RTA^E177D-HDEL^ while no cluster formation is observed in control experiments using eGFP-RTA^E177D^ lacking the ER-retention motif (Fig 1b). A qualitatively similar cluster development is observed for other human cell lines, SH-SY5Y and HEK-293T, even though there are visible differences in the details of their temporal evolution. Surprisingly, differentiated human THP1 macrophages do not show any GFP clusters at the cell surface throughout the experiment.

We also investigate cluster formation in the mouse cell lines. As can be seen in Fig 1c, the mouse fibroblast cell lines L929 and MEF exhibit a KDELR clustering similar to their human counterparts. Moreover, the macrophage cell lines IC21 and RAW-Blue likewise lack a KDELR clustering phenotype; both cell lines do not respond either to eGFP-RTA^E177D^ or eGFP-RTA^E177D-HDEL^ treatment. Therefore, we conclude that receptor clustering at the cell surface is not species specific and there are generally two types of cells: with or without ligand-induced KDELR cluster accumulation at the PM. We note that in this study only HDEL has been used as a representative ER retention motif. Therefore, we cannot exclude that the KDEL motif or less abundant H/KDEL-like motifs can be predominantly used by macrophages for a proper ligand uptake. The KDELR clustering process in macrophages should be validated in the presence of other ER retention motifs (e.g. KDEL or RTDL) in future studies.

**Fig 2:**
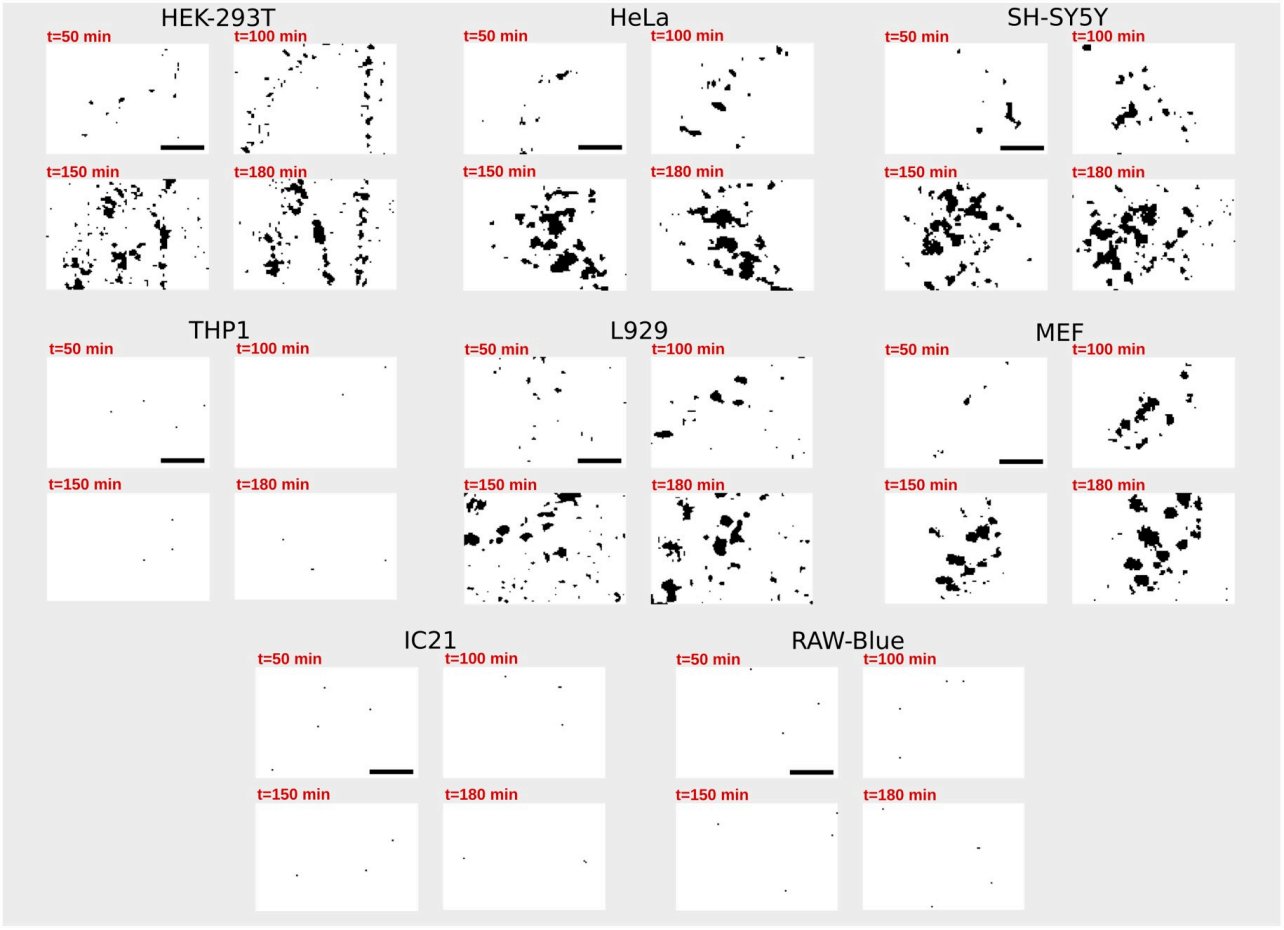
Evolution of the receptor clusters at the surface of various cell types. A randomly chosen region of the plasma membrane is shown at different time points. Scale bars, 4 *μ*m.

**Fig 3:**
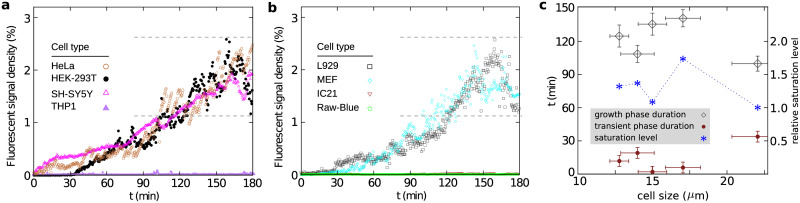
(a) Time evolution of the density of KDELR cargo signals at the surface of human cell lines after treatment with eGFP-RTA^E177D-HDEL^. The optimal signal-to-noise ratio, scaled by the cell periphery size, is shown as a function of time. The dashed lines show the fluctuation range of the signal intensity at the steady state. (b) Similar to panel (a) but for mouse cell lines. (c) Duration of transient and growth regimes and the mean relative saturation level of signal density (compared to the largest analyzed cells) in the steady state versus the cell size.

Next, we take a closer look at the time evolution of the amount of fluorescent signals (i.e. clusters of eGFP-RTA^E177D-HDEL^) at the cell surface. Fig 2 shows a randomly chosen part of the plasma membrane of various cell types. Here, for a better visualization of the receptor clusters, the gray-scale pixel intensity is converted to binary (black/white) data using an arbitrary threshold. Next, a precise procedure is applied for a quantitative evaluation of the amount of fluorescent signals (see the Materials and methods section for details). The cell lines showing cluster formation follow a similar temporal evolution as shown in Fig 3: the system initially remains almost inactive for a relatively short time (transient regime). Then, the clustering process speeds up with an increasing slope (exponential growth regime), until it eventually reaches a non-equilibrium steady state where the signal density fluctuates around a mean value due to the interplay between the stochastic loss of receptors via endocytosis and gain by exocytosis events (steady-state regime). Although the overall clustering process is similar in these cell types, the details of the temporal evolution of signal density differ from one cell to another. In order to clarify if the observed diversity is originated from the cell-size differences, we categorize the analyzed cells based on their sizes. To this aim, we approximate the mean cell size by the diameter of a circle with the same area as the cell. Fig 3c shows that there is no systematic dependence between the characteristics of the three regimes and the cell size. Presumably, the endocytosis and exocytosis rates are the main influential factors in the formation and growth of receptor clusters.

### Cluster size distribution

**Fig 4:**
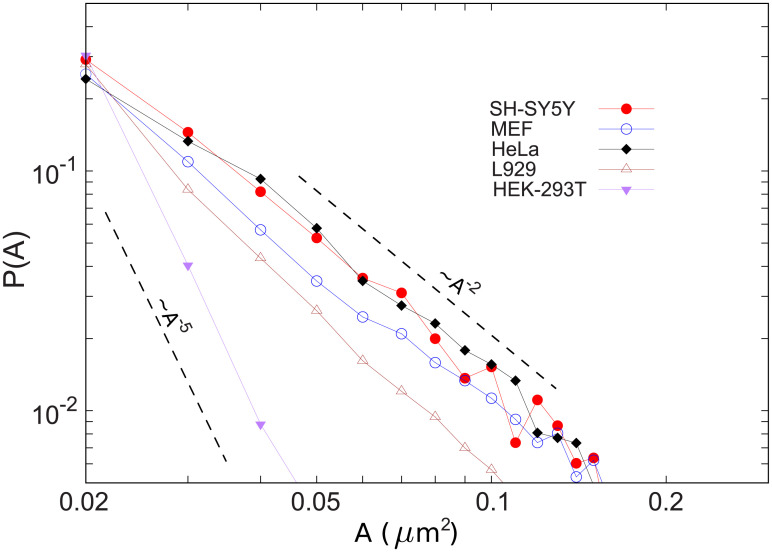
Cluster-size distribution of KDEL receptors at the surface of various cell types. The power-law exponent in the indicated cell lines varies from *α*≃2 for HeLa and SH-SY5Y cells to *α*≃5 for HEK-293T cells.

It was previously shown that the size distribution of the receptor clusters at the surface of HeLa cells decays as a power-law, indicating that there are preferred arrival sites at the plasma membrane [18]. Our analysis of the size of clusters in different cell types reveals that the cluster-size distribution in all cases nearly follows an algebraic form *P*(*A*)∼*A*^−*α*^ (Fig 4). The decay exponent is around *α* = 2 for HeLa and SH-SY5Y and *α* > 2 for MEF, L929, and HEK-293T cells. The exponents greater than 2 may evidence for a less efficient clustering process which prevents the formation of large clusters.

### KDELR mRNA levels are not correlated with cluster formation

**Fig 5:**
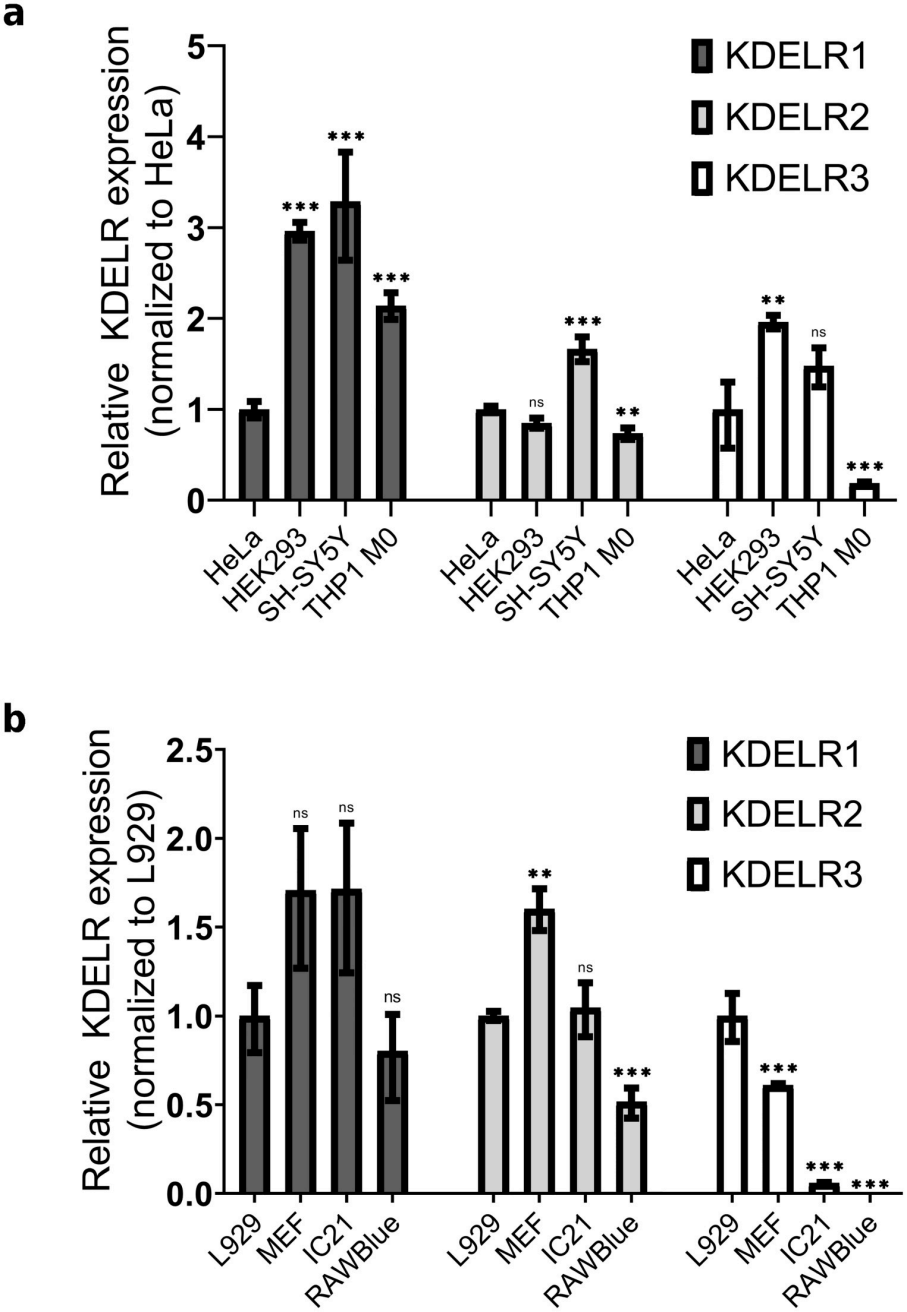
mRNA levels of three KDELR homologues in various (a) human and (b) mouse cell lines. Values in the indicated cell lines are scaled to the relative mRNA level of (a) HeLa and (b) L929 cells. Statistical significance is assessed by one-way ANOVA based on biological replicates and at sample sizes of n = 3 (***p ≤ 0.001; **p < 0.01; *p < 0.05; ns, not significant).

As a step towards identifying the responsible factors for the observed non-clustering phenotype of macrophage cell lines, we perform RT-qPCR experiments and compare the intracellular mRNA levels of three KDELR homologues to see if there are differences on the transcriptional level between macrophage-derived and other cells including fibroblast mouse cell lines (see Fig 5). The consistency of our data with the previous RT-qPCR results of HeLa [2] and HEK-293T and SH-SY5Y [7] cells verifies the reliability of our measurements. We find no correlation between the mRNA level of KDELR1 or KDELR2 and the observed clustering differences. mRNA levels of KDELR3 in all macrophage cell lines are significantly lower than in cluster-forming cells. However, the basal mRNA expression of KDELR3 is also extremely low compared to KDELR1/2 in all cell lines as shown in S1 Fig. Therefore, it is unlikely that the low mRNA level of KDELR3 is responsible for the missing receptor clustering phenotype seen in macrophages.

### Numerical approach

The results presented in the previous section raise the natural question: what is the difference between macrophage and other cell types? The formation of receptor clusters at the cell surface is a non-equilibrium process which ultimately reaches a steady state when the loss of receptors due to endocytosis is balanced with the gain by recycling them. We expect that an extremely high endocytosis or low exocytosis rate can practically prevent the formation of clusters. It is, however, unclear how large/small such rates have to be compared to those of cluster-forming cells. Since the adequate staining or detection of KDELRs via Western analysis or immunofluorescence is impossible due to a lack of suitable isoform-specific antibodies, we do not have the opportunity to biochemically quantify the amount of PM-localized KDELRs in different cell lines.

#### The model

**Fig 6:**
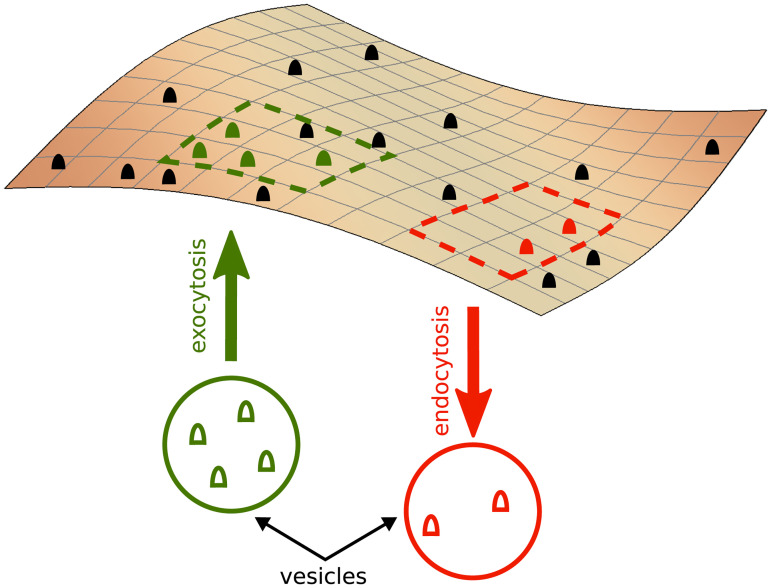
Schematic illustration of the receptor cycling model. Black, green, and red full symbols represent, respectively, the receptors which survive or will be added or eliminated in the next time step. Dashed lines indicate the affected zones by endo/exocytosis events.

To gain an insight into the interplay between endocytosis and exocytosis rates, we consider a stochastic process of internalization and vesicle arrival events to model the loss and gain of receptors at the cell surface. By ignoring the self-amplification effects for simplicity, we model the process in the following way: *(i) cell surface*: The membrane is modeled as a square lattice with periodic boundary conditions to mimic the closed cell surface. The lattice grid size is chosen to be 5 nm, which is in the order of magnitude of the KDEL receptor size [3, 25, 26]. Each lattice site is occupied by a liganded/unliganded receptor or remains empty (Fig 6). We consider a system of size *L* = 10 *μ*m, which is the approximate size of our smallest analyzed cells. *(ii) endocytosis events*: We assume that the positions of endocytosis events are uncorrelated in space and time. Thus, a random site is chosen as the center of the endocytosis event at each time step. The extent of the region affected by the endocytosis event is chosen randomly within 5 to 10 lattice sites around the center site, since the typical size of clathrin-coated vesicles is ∼50- 100 nm [27]. All the existing receptors within the affected region are eliminated (i.e. internalized). *(iii) exocytosis events*: The center of the target zone is randomly chosen from a multiple-peaked Gaussian distribution, with the peaks representing the places where microtubules approach the plasma membrane. The size of the arrival vesicle is chosen similarly to the endocytosis events. The random number of receptors carried by the arrival vesicle ranges from zero to the maximum capacity of the vesicle. These receptors are randomly distributed in the affected region upon availability of empty sites.

#### Formation and evolution of clusters

**Fig 7:**
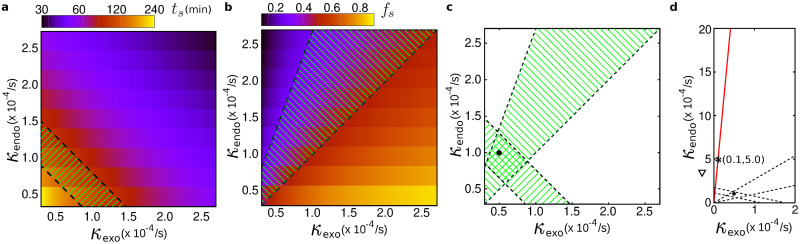
(a) Characteristic time *t*_*s*_ and (b) relative saturation level *f*_*s*_ in the (*κ*_endo_, *κ*_exo_) phase space. Single-hashed regions in panels (a) and (b) correspond to the values of *κ*_endo_ and *κ*_exo_ which result in *t*_*s*_ or *f*_*s*_ values experimentally observed for cluster-forming cells. (c) Double-hashed region shows the approximate extent of *κ*_endo_ and *κ*_exo_ rates in cell lines showing receptor clustering. The full circle represents the reference point (see text). (d) Similar to panel (c) but with different plot range. The solid line corresponds to the threshold line $\kappa_{\text{endo}}=\left(-1+1/f{\;}_{s}^{\;c}\right)\kappa_{\text{exo}}$, representing the unset of undetectable saturation level in experiments.

Starting from an initially empty membrane, surface density evolves and eventually reaches a non-equilibrium steady-state level with relatively large fluctuations. We characterize the steady state with the saturation time *t*_*s*_ (i.e. the characteristic time needed to reach $1-\frac{1}{e}$ fraction of the mean saturation density) and the saturation level *f*_*s*_ (defined as the fraction of the total receptors of the cell that are located on the cell surface, thus, *f*_*s*_ ∈ [0, 1]). Fig 6 shows how receptor clustering depends on the endocytosis *κ*_endo_ and exocytosis *κ*_exo_ rates (i.e. the effective receptor elimination and arrival rates in our Monte Carlo simulations). The characteristic time *t*_*s*_ varies by several orders of magnitude depending on the choice of *κ*_endo_ and *κ*_exo_. To determine the subset of the (*κ*_endo_, *κ*_exo_) phase space which is covered by cluster-forming cells, one can solve the master equation $\dot{f}=-\kappa_{\text{endo}}f+\kappa_{\text{exo}}\left(1-f\right)$ for the saturation level *f* to obtain its temporal evolution $f\left(t\right)=f{\;}_{s}+\left(f{\;}_{{}_{0}}-f{\;}_{s}\right)\;{\text{e}}^{-t/t_{s}}$, with $f{\;}_{{}_{0}}$ being the initial saturation level (taken to be zero in our simulations) and *t*_*s*_ = 1/(*κ*_endo_ + *κ*_exo_) being the characteristic time. Denoting the minimum and maximum duration of the exponential growth regime in experiments, respectively, with $t_{s}^{\text{min}}$ and $t_{s}^{\text{max}}$, then the lines $\kappa_{\text{endo}}+\kappa_{\text{exo}}=1/t_{s}^{\text{min}\;\text{(max)}}$ define the dashed lines in Fig 7a as the lower and upper borders of the endo/exocytosis rates. Furthermore, from the above master equation it can be seen that the relative saturation level is also given by the endo/exocytosis rates as *f*_*s*_ = *κ*_exo_/(*κ*_endo_ + *κ*_exo_). Fig 6b shows that the saturation level reduces with increasing the endocytosis and decreasing the exocytosis rates (upper-left corner). Assuming that *f*_*s*_ fluctuates in the experimental data within $\left[f{\;}_{s}^{\;\text{min}},f{\;}_{s}^{\;\text{max}}\right]$, we can determine the borders of the experimentally accessible region of the (*κ*_endo_, *κ*_exo_) phase space via the lines $\kappa_{\text{endo}}=\left(-1+\frac{1}{f{\;}_{s}^{\;\text{min}\;\text{(max)}}}\right)\kappa_{\text{exo}}$. While we know from our experiments that $f{\;}_{s}^{\;\text{max}}/f{\;}_{s}^{\;\text{min}}∼1.85$, the absolute value of $f{\;}_{s}^{\;\text{min}}$ or $f{\;}_{s}^{\;\text{max}}$ is unknown as we have no possibility to estimate the fraction of the total receptors of the cell that are located on the cell surface. Let us, for example, suppose that at most half of the receptors present at the plasma membrane in the steady state (i.e. $f{\;}_{s}^{\;\text{max}}=0.5$ and thus $f{\;}_{s}^{\;\text{min}}≃0.27$). This results in the two dashed lines in Fig 7b. Note that changing the values of $f{\;}_{s}^{\;\text{min}}$ or $f{\;}_{s}^{\;\text{max}}$ will change the slopes of the dashed lines in Fig 7b but does not qualitatively affect our following argument and conclusions. By combining the hashed regions in panels (a) and (b) of Fig 7, the double-hashed zone in Fig 7c is obtained which displays the approximate range of *κ*_endo_ and *κ*_exo_ rates for the cell lines showing cluster formation (i.e. HeLa, SH-SY5Y, HEK-293T, L929, and MEF cells). We choose a representative reference point in the bulk of this zone with *κ*_endo_ = 10^−4^/s and *κ*_exo_ = 0.5×10^−4^/s. Assuming that the relative saturation level *f*_*s*_ should be greater than a threshold (e.g. $f{\;}_{s}^{\;c}=0.02$) to be detectable in our experimental measurements, we calculate how far one should move from the reference point along the *κ*_endo_ or *κ*_exo_ axis to reach the undetectable threshold value $f{\;}_{s}^{\;c}$. We find that either an extremely high endocytosis rate *κ*_endo_ ∼ 24.5×10^−4^/s (approximately 25 times higher than the reference *κ*_endo_ value) or an extremely low exocytosis rate *κ*_exo_ ∼ 0.02×10^−4^/s (approximately 25 times lower than the reference *κ*_exo_ value) is required to have an undetectable receptor clustering saturation level. Using the former (latter) extreme rate and keeping the other rate fixed at its reference value, the system will quickly converge to the steady state in nearly seven minutes (a few seconds). While such extreme differences between the endo/exocytosis rates of macrophages and cluster-forming cells might be unexpected, it should be noted that any other combination of the rates satisfying $\kappa_{\text{endo}}=\left(-1+1/f{\;}_{s}^{\;c}\right)\kappa_{\text{exo}}$ (solid red line in Fig 7d) would also be a solution. For instance, a system with *κ*_endo_ = 5×10^−4^/s and *κ*_exo_ = 0.1×10^−4^/s (see the star shown in Fig 7d with a 5-fold difference with the coordinates of the reference point along each direction) converges to $f{\;}_{s}^{\;c}$ in nearly half an hour. Therefore, having only a few fold faster endocytosis rate together with a few fold slower exocytosis rate (compared to the reference point) can lead to an undetectable level of surface receptors at the steady state. Note that in the above discussion we have assumed that the surface density of receptors *n* is nearly the same for endo- and exocytosis. This assumption is critical at low densities because at least one receptor has to be present in order to trigger endocytosis. Since the probability of endocytosis depends on the size of local receptor clusters, the density of receptors in endo- and exocytosis might be different. We can account for this effect by introducing an effective endocytosis rate $\kappa_{\text{endo}}^{\prime }=n\;\kappa_{\text{endo}}$ containing the combined effects of internalization rate and surface occupation density. Then the above discussion remains valid for the effective endocytosis rate $\kappa_{\text{endo}}^{\prime }$.

#### Power-law decay of the cluster-size distribution

Towards understanding the origin of the diversity observed in the power-law exponent *α* of the cluster-size distribution in experiments (Fig 4), we consider a simple receptor aggregation process in which the preferential attachment of the newly arrived receptor to the existing clusters occurs with a given probability *β*. Indeed, *β* represents the efficiency of the directed transport to preferred docking sites on the membrane. Thus, the probability to attach to a cluster of size *A*_*i*_ is $P\left(A_{i}\right)=\frac{\partial A_{i}}{\partial t}=\beta \frac{A_{i}}{\sum_{j}A_{j}}$, where the sum runs over all existing clusters. Since $\sum_{j}A_{j}$ grows linearly with time, we have $\frac{\partial A_{i}}{\partial t}=\beta \frac{A_{i}}{t}$ leading to $A_{i}\left(t\right)={\left(\frac{t}{t_{i}}\right)}^{\beta }$, with *t*_*i*_ being the initiation time of cluster *i*. Then the cumulative probability that a cluster is smaller than *A* can be obtained as $F\left[A_{i}\left(t\right)<A\right]=F\left(t_{i}>\frac{t}{A^{1/\beta }}\right)=1-\frac{1}{A^{1/\beta }}$, where we assumed that *F*(*t*_*i*_) has a constant probability density, i.e. $F\left(t_{i}\right)=\frac{1}{t}$. Finally, the cluster-size distribution can be derived as $P\left(A\right)=\frac{\partial F[A_{i}\left(t\right)<A]}{\partial A}=A^{-(1+1/\beta )}$. This suggests that the power-law exponent *α* is related to the efficiency of the preferential attachment via *α* = 1 + 1/*β*. For *β* = 1, every arriving receptor chooses the preferred hot spots on the plasma membrane, leading to *α* = 2. However, *α* grows as the probability *β* to attach to the preferred docking sites on the membrane decreases. For instance, one obtains *α* = 5 for *β* = 1/4. An inefficient preferential attachment process slows down the growth rate of clusters and it is less likely that large clusters form on short time scales; as a result, the tail of the cluster-size distribution decays faster.

## Discussion and conclusions

In summary, we studied KDEL receptor clustering at the plasma membrane of different cell types from mouse and human origins via live cell imaging. We verified that the cluster formation is independent of species identity but there are cell-type differences: while no cluster formation is observed for macrophage cell lines from both species, other cell types such as fibroblast mouse cells develop clusters in a qualitatively similar manner.

Assuming that the KDELR internalization and recycling process exists in all cell lines including the macrophages, our numerical analysis suggests a few possible scenarios to prevent the formation of receptor clusters at macrophage cell surfaces: (i) the total number of receptors is too low in macrophages such that the arrival vesicles distribute too few receptors on the cell surface; (ii) one of the rates (either endocytosis or exocytosis) in macrophages differs considerably from that of cluster-forming cells, while the other rate behaves similarly to other cell types. According to this scenario, the endocytosis (exocytosis) events in macrophages should occur with at least a 25-fold higher (lower) rate compared to cluster-forming cells; (iii) a combination of faster endocytosis and slower exocytosis rates is responsible for the missing receptor clustering phenotype seen in macrophages. In this case, moderate differences compared to cluster-forming cells, such as a 5-fold higher endocytosis rate together with a 5-fold lower exocytosis rate, suffice to prevent the formation of receptor clusters at macrophage cell surfaces; and (iv) the surface density of KDELRs in macrophages may differ for endo- and exocytosis, compared to cluster-forming cells.

The first scenario is already disproved indirectly by our RT-qPCR results, verifying that the levels of KDELR1/2 mRNA in cluster-forming cell lines are similar to the cells forming no clusters. Although mRNA level of KDELR3 is lower in macrophages, we expect that this receptor type plays a rather insignificant role in general due to the extremely low expression level of KDELR3 compared to KDELR1/2 (see S1 Fig).

As a part of the immune system, macrophages serve as professional phagocytotic cells and are specialized to detect and quickly eliminate pathogen particles such as cell debris or bacteria [28, 29]. The internalization rate of macrophages strongly depends on the type of particle, ranging from a half-life of a couple of seconds for fast phagocytotic events [30] to minutes or even hours for ligand/receptor endocytosis [31, 32]. There are however other cell types which perform clathrin-driven receptor endocytosis with a similar rate and internalize surface bound ligands in a few minutes [33–35].

An extremely high endocytosis rate implicates a high ligand uptake rate. Subsequently, intracellular KDELR/ligand signals should be visible over the long imaging period of 3 h. We, however, observe no GFP signals in macrophage cell lines. Rapid lysosomal degradation and the associated deprivation of the GFP fluorescence in the model cargo is also unlikely, because the interaction with KDELRs should mainly prevent the ligand transport in this organelle and foster its targeted retrograde transport into the ER.

A very low exocytosis rate of KDELRs to the PM could be also a possible explanation for the observed non-clustering phenotype. Nevertheless, the quantification of PM-localized KDELRs is so far impossible due to the lack of suitable antibodies for immunofluorescence studies. Moreover, overexpression of tagged KDELRs is in principle possible but the overload of the natural ER retention system may dramatically affect the results leading to misinterpretations.

A complete absence of PM-localized KDELRs could be another explanation for the observed phenotype. It is possible that macrophage cell lines have an active mechanism to prevent PM-transport of KDELRs or lacking specific cellular components (i.e. proteins and/or signaling pathways) required for proper cell surface transport or ligand binding recognition. In such a case, macrophages cannot respond to the external applied ligand and receptor clusters do not form.

Concerning the biological relevance of having different clustering dynamics at different cell types, if there is a signaling pathway involved in the receptor clustering process one could speculate that a certain cluster size threshold has to be reached to activate this signaling pathway or to trigger the subsequent cluster internalization from the PM. Additionally, the cluster size could reflect the differences in the pool of PM-localized KDELR homologues, where the amount and/or type of receptor define the maximum cluster size and endocytosis rate. The differences in cluster dynamics could also correlate with the total amount of KDELRs on the PM and determine the temporal development of clusters and strength of intrinsic cellular responses, possibly specializing subsets of cell types for a specific KDELR-ligand dependent response.

Our results thus call for systematic studies to better understand the internal mechanisms of receptor clustering and to clarify the differences between macrophages and other cell types, e.g. in their endo/exocytosis rates or in the total amount of PM-localized KDELRs. To this aim, better antibodies and tools are required for quantitative comparisons. Understanding why macrophages do not form ligand/receptor clusters could also shed light on how these cells achieve an efficient immune response and interact with tissue cells containing KDELRs at their cell surface.

## Supporting information

S1 Fig Relative KDELR1-3 mRNA levels. mRNA levels were determined with RT-qPCR experiments in (a) human cells (HeLa, HEK-293T, SH-SY5Y, and THP1) and (b) mouse cells (L929, MEF, IC21, and RAW-Blue). mRNA levels were normalized to the human or mouse reference gene GAPDH and KDELR1 mRNA level was set to 100%.
(EPS)

S1 Table Primer sequences used in this study. (PDF)

S2 Table Nucleotide sequences for qPCR primers (ordered from Invitrogen). (PDF)

S3 Table Overview of the basic characteristics of the cell lines used in this study. (PDF)
