# Supplementary material for: Cell-type-specific differences in KDEL receptor clustering in mammalian cells
Source: PLoS One. 2020 Jul 9;15(7):e0235864. doi: 10.1371/journal.pone.0235864 (PMC7347126; doi:10.1371/journal.pone.0235864)
Supplement: S1 Table — (PDF) [file pone.0235864.s002.pdf]

Supplementary Table S1. Primer sequences used in this study

Primer name	5'-3' sequence
5'-RTA^{E177D}	GAATTCGGATCCATGATATTCCCCAAACAATACCCAATTATAAACTTTACC
3'-RTA^{E177D}	AAGCTTGTCGACTTAATGATGATGATGATGATGAAACTGTGACGATGGTGGAGGTGC
3'-RTA$^{E177D\text{-}HDEL}$	AAGCTTGTCGACTTACAGTTCATCATGATGATGATGATGATGATGAAACTGTGACGATGGTGGAGGTGC
