# Supplementary material for: Cell-type-specific differences in KDEL receptor clustering in mammalian cells
Source: PLoS One. 2020 Jul 9;15(7):e0235864. doi: 10.1371/journal.pone.0235864 (PMC7347126; doi:10.1371/journal.pone.0235864)
Supplement: S2 Table — (PDF) [file pone.0235864.s003.pdf]

Supplementary Table S2. Nucleotide sequences for qPCR primers (ordered from Invitrogen)

Target	Forward	Reverse
Human KDELR1 NC 000019	CACAGCCATTCTGGCGTTCCTG	CCATGAACAGCTGCGGCAAGAT
Human KDELR2 NC 000007	CTGGTCTTCACAACTCGTTACCTGGATC	CAGGTAGATCAGGTACACTGTGGCATAGG
Human KDELR3 NC 000022	CTTCTGGTCCCAGTCATTGGCCT	GGGGCAGGATAGCCACTGATTCC
Human GAPDH NC 000012	TTCGACAGTCAGCCGCATCT	GCCCAATACGACCAAATCCGTT
Mouse KDELR1 NC 000073	GTGGTGTTCACTGCCCGATA	AACTCCACCCGGAAAGTGTC
Mouse KDELR2 NC 000071	TGGTCTTCACGACTCGCTAC	AGGTACACCGTGGCATAGGA
Mouse KDELR3 NC 000081	CTTCATCTCCATCTACAACACAGTG	CTCCAGCCGGAATGTGTCAT
Mouse GAPDH NC 000072	GAGAGTGTTTCCTCGTCCCG	TCCCGTTGATGACAAGCTTCC
