# Supplementary material for: Cell-type-specific differences in KDEL receptor clustering in mammalian cells
Source: PLoS One. 2020 Jul 9;15(7):e0235864. doi: 10.1371/journal.pone.0235864 (PMC7347126; doi:10.1371/journal.pone.0235864)
Supplement: S3 Table — (PDF) [file pone.0235864.s004.pdf]

Supplementary Table S3. Overview of the basic characteristics of the cell lines used in this study

Name	Morphology	Tissue	Organism	Source
HeLa	epithelial	cervix	Homo sapiens	ATCC
HEK-293T	epithelial	embryonic kidney	Homo sapiens	Invitrogen
SH-SY5Y	neuroblast	bone marrow	Homo sapiens	Sigma
THP1	monocyte	peripheral blood	Homo sapiens	ATCC
MEF	fibroblast	embryo fibroblast	Mus musculus	Gift from Prof. Aránzazu del Campo
L929	fibroblast	subcutaneous connective tissue	Mus musculus	Gift from Prof. Aránzazu del Campo
RAW-Blue	macrophage	Abelson murine leukemia virus-induced tumor	Mus musculus	Invitrogen
IC21	macrophage	peritoneum	Mus musculus	Gift from Prof. Gernot Geginat
